# Supplementary material for: Complex‐centric proteome profiling by SEC‐SWATH‐MS
Source: Mol Syst Biol. 2019 Jan 14;15(1):e8438. doi: 10.15252/msb.20188438 (PMC6346213; doi:10.15252/msb.20188438)
Supplement: Supplementary file 8 — Dataset EV7 [file MSB-15-e8438-s008.zip › feature_plots_string/Q13405.pdf]

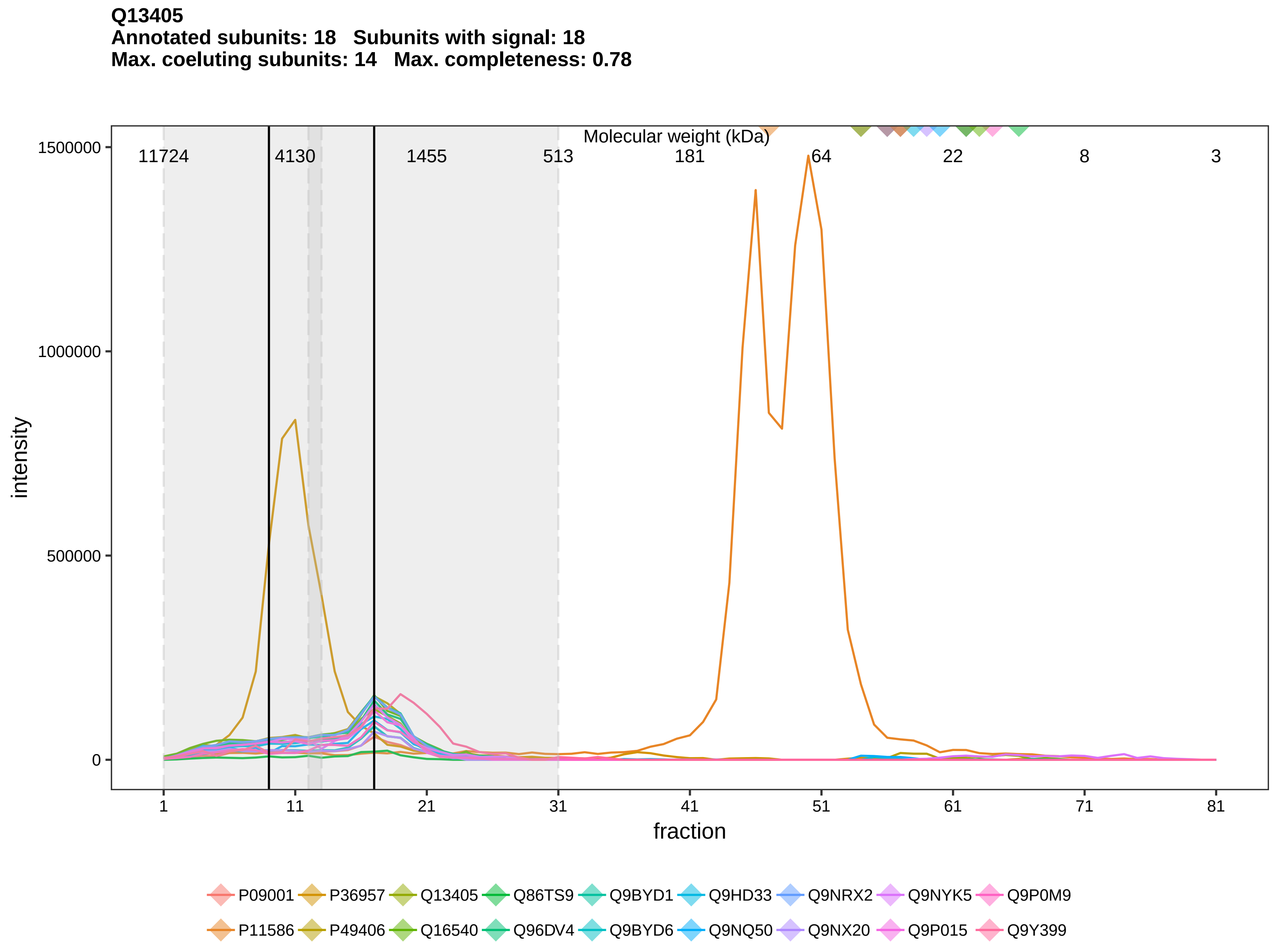
Q13405
Annotated subunits: 18 Subunits with signal: 18
Max. coeluting subunits: 14 Max. completeness: 0.78
Molecular weight (kDa)
11724
4130
1455
513
181
64
22
8
3
intensity
1500000
1000000
500000
0
fraction
1
11
21
31
41
51
61
71
81
P09001
P11586
P36957
P49406
Q13405
Q16540
Q86TS9
Q96DV4
Q9BYD1
Q9BYD6
Q9HD33
Q9NQ50
Q9NRX2
Q9NX20
Q9NYK5
Q9P015
Q9P0M9
Q9Y399
